# Irreversibility analysis of electromagnetic hybrid nanofluid for Cattaneo–Christov heat flux model using finite element approach

**DOI:** 10.1038/s41598-023-31445-7

**Published:** 2023-03-15

**Authors:** Muhammad Amer Qureshi

**Affiliations:** 1 PYP-Mathematics, College of General Studies, King Fahd University of Petroleum and Minerals, Dhahran, Saudi Arabia; 2 Interdisciplinary Research Center for Hydrogen and Energy Storage, King Fahd University of Petroleum and Minerals, Dhahran, Saudi Arabia

**Keywords:** Mathematics and computing, Nanoscience and technology

## Abstract

To get a better heat transmission capacity of ordinary fluids, new hybrid nanofluids (HNFs) with a considerably greater exponent heat than nanofluids (NFs) are being used. HNFs, which have a greater heat exponent than NFs, are being applied to increase the HT capacities of regular fluids. Two-element nanoparticles mixed in a base fluid make up HNFs. This research investigates the flow and HT features of HNF across a slick surface. As a result, the geometric model is explained by employing symmetry. The technique includes nanoparticles shape factor, Magnetohydrodynamics (MHD), porous media, Cattaneo–Christov, and thermal radiative heat flux effects. The governing equations are numerically solved by consuming a method known as the Galerkin finite element method (FEM). In this study, H_2_O-water was utilized as an ironic, viscous improper fluid, and HNF was investigated. Copper (Co) and Titanium Alloy (Ti_6_Al_4_V) nanoparticles are found in this fluid. The HT level of such a fluid (Ti_6_Al_4_V-Co/H_2_O) has steadily increased in comparison to ordinary Co-H_2_O NFs, which is a significant discovery from this work. The inclusion of nanoparticles aids in the stabilization of a nanofluid flowing and maintains the symmetry of the flow form. The thermal conductivity is highest in the boundary-lamina-shaped layer and lowest in sphere-shaped nanoparticles. A system's entropy increases by three characteristics: their ratio by fractional size, their radiated qualities, and their heat conductivity modifications. The primary applications of this examination are the biological and medical implementations like dental and orthopedic implantable devices, as well as other devices such as screws and plates because they possess a favorable set of characteristics such as good biomaterials, corrosion resistance and wear, and great mechanical characteristics.

## Introduction

Nanofluids (NFs) have been considered as a potential different fluid solution for enhancing the competence and efficacy of current systems in manufacturing, commercial, and residential contexts. Numerous benefits of increased thermal system efficiency include decreased environmental impact, decreased energy use, and lower prices. The appropriateness of NFs for use in present systems has recently been assessed in terms of cost and environmental impact by utilising sustainability approaches. Thermal studies are one of its most important applications. The energy consumption of thermal systems is essential in the global environment. Several readings have been shown to increase the performance of thermal systems based on these elements, including the employment of various resources, produced liquids, process proposals, and the integration of newfangled information for clean energy building, resulting in an optimal explanation. Increasing the heat surface area of thermal convert to recover their current performance is one of the most investigated solutions; however, this modification results in the material buildup and an increase in production cost. In order to ensure long-term technical development, Bretado et al.^1^ underlined the expansion of NFs in thermal applications and offered a review of their benefits and zones of opportunity. Waste heat recovery, which tries to recover energy losses as heat, work, or power, was researched by Olabi et al.^2^. They claim that NFs are recently developed high-performance heat transfer fluids. Three crucial factors identified by Wang et al.^3^ have an impact on the use of mono and hybrid NFs in heat pipes. Consistency, thermal conductivity, and viscosity. The application of heat transfer growth or inhibition, as well as the usage of NFs in a variety of heat pipe categories, is described. Machine learning is explored in the context of NFs (thermal conductivity and dynamic viscosity) and NF-charged heat pipes. Current developments in NF thermal characteristics and applications in a variety of engineering fields, ranging from NF-medicine to renewable energy, were examined by Eid^4^. The latter has seen some major advancements in flexibility and momentum, which have an impact on military and shield technologies. As a result, specialised NF applications in space research, solar energy, NF-medicine, temperature exchangers, heat pipes, and electronics freezing have been researched and made available. Gupta et al.^5^ examined the current advancements in NF in solar collectors and how it is employed nowadays. They discovered that using a premium heat transfer fluid with outstanding thermal physical properties, such as high thermal conductivity, is the most efficient way to increase the performance of a solar energy system, and NF is the best option for doing so. According to Salilih et al.^6^, the use of NF resulted in decreased heat of liquid leaving the condenser, increasing the solar scheme's efficacy.

Jana et al.^7^ largely addressed hybrid nanofluid (HNF), a modern class of NF created by suspending separate multiple NFs in the base NFs. Unexpectedly, the thermal characteristics can be increased by the creation of a small portion of metal nanotubes or nanoparticles within the NFs of an oxide or metal that are already present in a base liquid. Improved thermal conductivity, stability, corrected HT, positive impacts of each suspension, and combined nanomaterial influence are only a few of the benefits of HNFs. With higher operational efficiencies than NFs, HNFs are used in almost all HT applications, including welding, defense, temperature pipe, biomedical, boats, and space planes. Other applications include generator freezing, coolant in machining, thermal capacity, electronic cooling, reheating and cooling in homes, vehicle thermal management or motor freezing, modernizer freezing, atomic structure freezing, refrigeration, medication saving, and vehicle thermal management or motor freezing. These good properties drew researchers' attention to the HNF in the context of HT difficulties in daily living. Khan et al.^8^ presented a proportional investigation of HT and friction drag in the flow of numerous HNFs achieved by the associated magnetic field and nonlinear radiation. Xiong et al.^9^ reviewed the application of HNFs in solar energy collectors. While Yaseen et al.^10^ reviewed the role of HNFs in HT. Sathyamurthy et al.^11^ documented an experimental investigation on freezing the photovoltaic board utilizing HNFs. Bakhtiari et al.^12^ presented stable HNFs and advanced a novel association for HT. Xuan et al.^13^ studied thermo-economic presentation and compassion examination of ternary HNFs. Said et al.^14^ gathered HT, entropy generation, and economic and ecological examinations of linear Fresnel indicators utilizing HNFs. Jamshed et al.^15^ introduced a computational setting effort of the Cattaneo–Christov heat flux model (CCHFM) based on HNFs. Ma et al.^16^ considered the effect of surfactants on the rheological performance of HNF and HT ownership. Chu et al.^17^ modeled a study of magnetohydrodynamics utilizing HNFs flow between two endless corresponding platters with atom form possessions. Şirin^18^ investigated the presentation of cermet apparatuses in the rotating of HNFs wounding settings. Jamei et al.^19^ estimated the thickness of HNFs for current dynamism application. Bilal et al.^20^ used the degenerate electro-osmotic EMHD HNFs over the micro-passage.

A porous media model (PMM), often recognized as a porous material, is one that contains pores (vacuums). The "matrix" or "frame" refers to the thin part of the fabric. A fluid is generally injected into the pores (fluid or fume). Although the skeleton fabric is typically solid, systems together with foams may enjoy the perception of a porous media model (PMM). Jamshed et al.^21^ used PMM in solar aircraft joining tangent HNFs as a solar heat application. Shahzad et al.^22^ formulated a comparative mathematical study of HT using the PMM in HNFs. Parvin et al.^23^ presented the numerical conduct of 2D-Magneto double-diffusive convection flow of HNF over PMM. Faisal, et al.^24^ indicated the raising of heat effectiveness of solar water‐pump utilizing HNFs over PMM. Banerjee and Paul^25^ reviewed the most recent studies and development with the applications of PM combustion. Zou et al.^26^ modeled an explicit system of stone heat in the PM model for pebble-bed devices. Lee et al.^27^ proposed PMM substantiation with stress drip dimensions. Talbi et al.^28^ analyzed a solution for longitudinal quivering of a fluctuating pile based on PMM on a convective flowing model.

Alizadeh et al.^29^ took into consideration a device studying technique for the calculation of transference and thermodynamic methods in metaphysics structures HT in HNFs flow in PMM. Rashed et al.^30^ recommended a non-homogenous HNF for three-D convective flow in enclosures full of heterogeneous PMM. The investigation of the magnetic appearances and behavior of electrically conducting liquids is known as magnetohydrodynamics (MHD). Plasmas, melted metals, salty water, and electrolytes are illustrations of MHD. Recently, many investigations are appeared using this setting practically in HNFs. Alghamdi et al.^31^ utilized MHD HNFs flow encompassing the medicine over a blood artery. Zainal et al.^32^ analyzed MHD HNFs flow over an extending/dwindling pane with quadratic velocity. Abbas et al.^33^ modeled improper investigation of motivated MHD of HNFs flow over a nonlinear extending cylinder. Waqas et al.^34^ impacted of MHD radiated flow of HNF over a revolving disk. Shoaib et al.^35^ provided a numerical examination of three-D MHD HNFs over a revolving disk in the incidence of heat electricity with Joule reheating and viscous degeneracy possessions using the Lobatto method. Tian et al.^36^ investigated 2D and 3-d shapes of fins and their possessions on the heat sink performance of MHD HNF with slide and non-slip float. Gul et al.^37^ studied a couple of slides impacted withinside the MHD HNF float with Cattaneo–Christov heat flux and autocatalytic biochemical response. Ashwinkumar et al.^38^ considered HT in MHD HNFs flow over two diverse geometries. Abderrahmane et al.^39^ formulated MHD HNFs over HT and entropy generation in a 3D revolving tube. Salmi et al.^40^ studied a numerical case of non-Fourier heat and mass transfer in incompletely ionized MHD HNFs.

The heat transfer in viscoelastic float resulting from an exponentially stretched sheet is defined through the Cattaneo–Christov warmth flux model (CCHFM). The major factors of this study may be summarized as follows: When related to a viscous fluid, the hydrodynamic boundary layer in the viscoelastic fluid is thinner. Venkata et al.^41^ considered CCHFM on sloping MHD over nonlinear overextended flow. Haneef et al.^42^ utilized CCHFM and HT in HNFs rheological liquid in the attendance of mass transfer. Yahya et al.^43^ employed CCHFM on Williamson Sutterby NF transportation, which is produced by an extending superficial with a convective boundary. Eswaramoorthi et al.^44^ engaged CCHFM in 3D plow of a plate with nonlinear heat energy. Tahir et al.^45^ enhanced the current appearances of viscous NF flow with the induction of CCHFM. Ali et al.^46^ proposed CCHFM for assorted convection flow owing to the revolving disk with slide possessions. Ullah et al.^47^ suggested a numerical attitude to read melting and initiation energy occurrence on the influenced fleeting HNF with the application of CCHFM. Zuhra et al.^48^ gave a numerical analysis of CCHFM HNFs by Lavenberg–Marquard back propagated neural networks. Sadiq et al.^49^ modeled the HT because of CCHFM. Vinodkumar et al.^50^ joined the CCHFM HNFs that affected MHD flow via an extending slip in a PMM.

The no-slip condition is the acknowledged boundary condition for a fluid over a solid surface. The slip boundary condition (SBC) proposed by Navier^51^ is one in which the slide velocity is compared to the clip stress. Alzahrani et al.^52^ studied the effect of heat contamination on HT in-plane walls themed to SBC. Pérez-Salas et al.^53^ presented an approximate analytical outcome for the fluid flow of a Phan-Thien-Tanner with SBC. Wang et al.^54^ solved the problem of SBC by boundary-lattice Boltzmann scheme. Arif et al.^55^ analyzed SBC of Non-Newtonian rheology of lubricant. Dhifaoui^56^ illustrated a weak solution for the outside static Stokes equations with SBC. Zeb et al.^57^ proposed the SBC on Non-Newtonian Ferrofluid over an extending slip. There are many studies^58–60^ probed the problem of slippage velocity in the flow model. It had a prominent effect in clarifying this effect on the movement of the fluid and its temperature.

This looks at objectives to fill a familiarity hole withinside the flow and warmth transfer of a radiated Casson HNF with a variable thermal conductivity because the temperature rises, primarily based totally on the literature. The Tiwari and Das NF versions can be used to mathematically version the NF flow. Copper (Cu) and Titanium Alloy (Ti_6_Al_4_V) are the two types of HNFs used in this study. Entropy generation data for HNFs used in this study was analyzed to identify the impact on the process. The HNF's governing equations will be translated into ODEs using an appropriate similarity conversion. ODEs will be created, and the Galerkin finite element method (FEM) will be utilized to numerically resolve them using appropriate governing parameter values. The numbers are going to be represented graphically, with additional discussion. The impacts of particle shapes, thermal radiated flow, slippery velocity, and convective slip boundary limitations are investigated during this research.

## Governing equations and material

**Figure 1:**
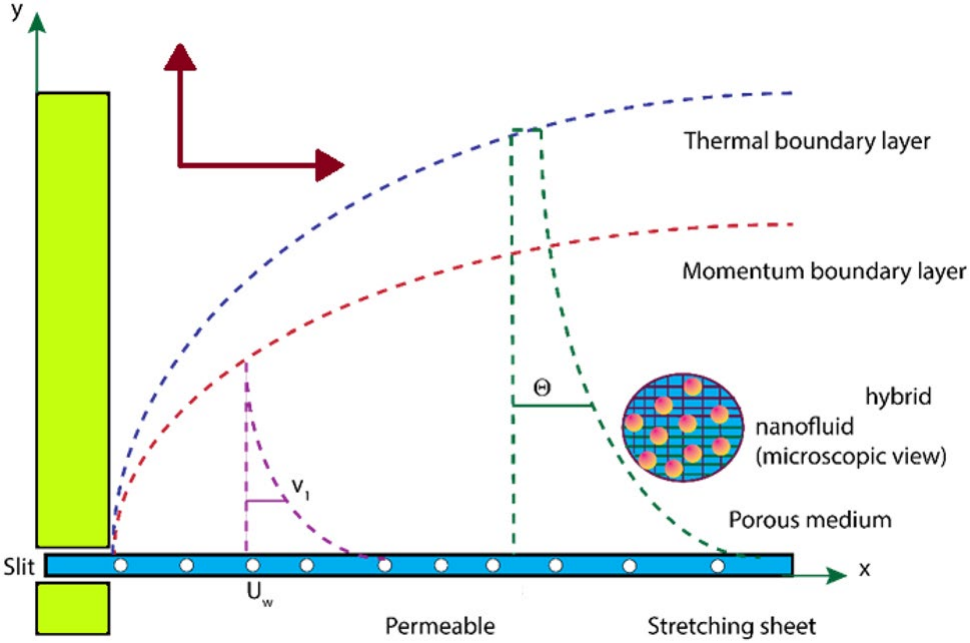
Flow model description.

Consider the 2D steady symmetric flow of magnetized hybrid nano-liquid over a stretchable surface examining the characteristics of Cattaneo–Christov heat flux across the fluid flow in *x*-path. The *xy*-coordinate system is taken where the *x*-axis is along the path of the flowing, and *y*-axis is normal to the flowing with a stretching rapidity ${U}_{w}=qx$, as depicted in Fig. 1. Magnetic field, has potently ${B}_{0}$ is applied. Further, ${\mathrm{\yen }}_{w}\left(x,0\right)={\mathrm{\yen }}_{\infty }+{q}^{*} x,$ is disconnected surface temperature, for propriety, it is shown as consistent at $x=0$. Here $q,$ ${q}^{*},$ ${\mathrm{\yen }}_{w}$ and ${\mathrm{\yen }}_{\infty }$ address the unique growth rate, the pace of temperature variety, and the temperature of the surface and encompasses individually.

The fundamental (geometrical) curving prototype is drawn in Fig. 1:

The ensuing standards, together with the requirements, be relevant to the stream framework: 2-D laminar steady flow, phase flow model, HNF, permeable medium, MHD, viscous dissipation, Thermal radiative heat flux, Cattaneo–Christov heat flux, joule heating, porousness elongated surface.

The governing equations and associated boundary conditions for hybrid nanofluid flowing are given in^61^ in consideration of the suggested assumptions.

1 $${({\Lambda }_{1})}_{\mathrm{x}}+{({\Lambda }_{2})}_{y}=0,$$

2 $${\Lambda }_{1}{({\Lambda }_{1})}_{\mathrm{x}}+{\Lambda }_{2}{({\Lambda }_{1})}_{\mathrm{y}}=\frac{1}{{\rho }_{hnf}}\left[{{\mu }_{hnf}({\Lambda }_{1})}_{\mathrm{yy}}+{\mu }_{hnf}{\Lambda }_{1}-{\sigma }_{hnf}{B}^{2}{\Lambda }_{1}\right],$$

3 $${\Lambda }_{1}{\mathrm{\yen }}_{\mathrm{x}}+{\Lambda }_{2}{\mathrm{\yen }}_{\mathrm{y}}=\frac{1}{(\rho {C}_{p}{)}_{hnf}}\left[{k}_{hnf}\left({\mathrm{\yen }}_{\mathrm{yy}}\right)-{({q}_{r})}_{\mathrm{y}}+{\left({({\Lambda }_{1})}_{\mathrm{y}}\right)}^{2}+{\sigma }_{hnf}{B}^{2}{\Lambda }_{1}^{2}-\Upsilon \left[{\Lambda }_{1}{({\Lambda }_{1})}_{\mathrm{x}}{\mathrm{\yen }}_{\mathrm{x}}+{\Lambda }_{2}{({\Lambda }_{2})}_{\mathrm{y}}{\mathrm{\yen }}_{\mathrm{y}}+{\Lambda }_{1}{({\Lambda }_{2})}_{\mathrm{x}}{\mathrm{\yen }}_{\mathrm{y}}+{\Lambda }_{2}{({\Lambda }_{1})}_{\mathrm{y}}{\mathrm{\yen }}_{\mathrm{x}}+{\Lambda }_{1}^{2}{\mathrm{\yen }}_{\mathrm{xx}}+{\Lambda }_{2}^{2}{\mathrm{\yen }}_{\mathrm{yy}}+2{\Lambda }_{1}{\Lambda }_{2}{\mathrm{\yen }}_{\mathrm{xy}}\right] \right].$$

Jamshed et al.^21^ gave the related boundary constraints:

$${\Lambda }_{1}(x,0)={U}_{w}+{N}_{\Lambda }{({\Lambda }_{1})}_{y}, {\Lambda }_{2}(x,0)={V}_{\Lambda }, -{k}_{\Lambda }\left({\mathrm{\yen }}_{\mathrm{y}}\right)={h}_{\Lambda }({\mathrm{\yen }}_{w}-\mathrm{\yen }$$)

4 $${\Lambda }_{1}\to 0, \mathrm{\yen }\to {\mathrm{\yen }}_{\infty } as y\to \infty .$$

where, flow velocity ($\overleftarrow{\Lambda }=[{\Lambda }_{1}(x,y),{\Lambda }_{2}(x,y),0]$), temperature ($\mathrm{\yen }$),magnetic field strength ($B$), porosity ($k$), thermal radiation (${q}_{r}$), thermal relaxation time $\left(\Upsilon\right),$ slip length (${N}_{\Lambda }$), heat transfer coefficient $\left({h}_{\Lambda }\right),$ surface permeability ${(V}_{\Gamma }),$ thermal conductivity of the surface (${k}_{\Omega }$).

The equations in Table 1 summarize NF and HNF variables of the material^62–64^.

**Table 1:** Thermo-physical features of nanofluids and hybridnanofluids.

Features	Nanofluid	Hybrid nanofluid
Viscosity $(\mu )$	${\mu }_{nf}={\mu }_{f}(1-\phi {)}^{-2.5}$	${\mu }_{hnf}$ = ${\mu }_{f}(1-{\phi }_{Co}{)}^{-2.5}(1-{\phi }_{TA}{)}^{-2.5}$
Density $(\rho )$	${\rho }_{nf}=\left(1-\phi \right){\rho }_{f}-\phi {\rho }_{s}$	${\rho }_{hnf}$ = [$(1-{\phi }_{TA})\{(1-{\phi }_{Co}){\rho }_{f}+{\phi }_{Co}{\rho }_{{p}_{1}}\}]$ + ${\phi }_{TA}{\rho }_{{p}_{2}}$
Heat capacity $(\rho {C}_{p})$	$(\rho {C}_{p}{)}_{nf}=(1-\phi )(\rho {C}_{p}{)}_{f}-\phi (\rho {C}_{p}{)}_{s}$	$(\rho {C}_{p}{)}_{hnf}$ = $[(1-{\phi }_{TA})\{(1-{\phi }_{Co})(\rho {C}_{p}{)}_{f}+{\phi }_{Co}(\rho {C}_{p}{)}_{{p}_{1}}\}]+{\phi }_{TA}(\rho {C}_{p}{)}_{{p}_{2}}$
Thermal conductivity $(\kappa )$	$\frac{{\kappa }_{nf}}{{\kappa }_{f}}=\left[\frac{({\kappa }_{s}+2{\kappa }_{f})-2\phi ({\kappa }_{f}-{\kappa }_{s})}{({\kappa }_{s}+2{\kappa }_{f})+\phi ({\kappa }_{f}-{\kappa }_{s})}\right]$	$\frac{{\kappa }_{hnf}}{{\kappa }_{gf}}=\left[\frac{({\kappa }_{{p}_{2}}+2{\kappa }_{gf})-2{\phi }_{TA}({\kappa }_{gf}-{\kappa }_{{p}_{2}})}{({\kappa }_{{p}_{2}}+2{\kappa }_{gf})+{\phi }_{TA}({\kappa }_{gf}-{\kappa }_{{p}_{2}})}\right],$ $\frac{{\kappa }_{gf}}{{\kappa }_{f}}=\left[\frac{({\kappa }_{{p}_{1}}+2{\kappa }_{f})-2{\phi }_{Co}({\kappa }_{f}-{\kappa }_{{p}_{1}})}{({\kappa }_{{p}_{1}}+2{\kappa }_{f})+{\phi }_{Co}({\kappa }_{f}-{\kappa }_{{p}_{1}})}\right]$
Electrical conductivity $(\sigma )$	$\frac{{\sigma }_{nf}}{{\sigma }_{f}}$=$\left[1+\frac{3(\frac{{\sigma }_{s}}{{\sigma }_{f}}-1)\phi }{(\frac{{\sigma }_{s}}{{\sigma }_{f}}+2)-(\frac{{\sigma }_{s}}{{\sigma }_{f}}-1)\phi }\right]$	$\frac{{\sigma }_{hnf}}{{\sigma }_{f}}$ = $\left[1+\frac{3(\frac{{\phi }_{Co}{\sigma }_{{p}_{1}}+{\phi }_{TA}{\sigma }_{{p}_{2}}}{{\sigma }_{f}}-({\phi }_{Co}+{\phi }_{TA}))}{(\frac{{\phi }_{Co}{\sigma }_{{p}_{1}+{\phi }_{TA}{\sigma }_{{p}_{2}}}}{({\phi }_{Co}+{\phi }_{TA}){\sigma }_{f}}+2)-(\frac{{\phi }_{Co}{\sigma }_{{p}_{1}}+{\phi }_{TA}{\sigma }_{{p}_{2}}}{{\sigma }_{f}}-({\phi }_{Co}+{\phi }_{TA}))}\right]$

Where, nano-sized particle fractional volume ($\phi$), fluid and density $({\rho }_{f }\& {\rho }_{s})$, fluid and particle heat capacity $(({C}_{p}{)}_{f} \& \left({C}_{p}{)}_{s}\right),$ fluid and particle thermal conductivity $\left({\kappa }_{f} \& {\kappa }_{s}\right),$ hybrid nano-sized particle fractional volume (${\phi }_{hnf}={\phi }_{Co}+{\phi }_{TA}$), viscidness of the hybrid nanoliquid $({\mu }_{hnf})$, density of the hybrid nanoliquid $({\rho }_{hnf})$, heat capacitance of the hybrid nanoliquid $(\rho ({C}_{p}{)}_{hnf})$, thermal conductance of hybrid nanoliquid $\left({\kappa }_{hnf}\right).$

Further, ${\rho }_{{p}_{1}}$, ${\rho }_{{p}_{2}}$, $({C}_{p}{)}_{{p}_{1}}$, $({C}_{p}{)}_{{p}_{2}}$, ${\kappa }_{{p}_{1}}$ and ${\kappa }_{{p}_{2}}$ are the density, specific-heat capacitance, and thermal conductance of the nanomolecules.

In Table 2 (^65–67^) of analysis, substantial features of the primary fluid of the water are described.

**Table 2:** Fabricated materials with thermo-physical attributes.

Thermophysical	$\rho (\mathrm{kg}/{\mathrm{m}}^{3})$	${c}_{p} (\mathrm{J}/\mathrm{kgK})$	$k (\mathrm{W}/\mathrm{mK})$	$\sigma (\mathrm{S}/\mathrm{m})$
Water (H_2_O)	997.1	4179	2.613	0.05
Copper (Cu)	8933	385.0	401.00	5.96 × 10^7^
Titanium alloy (Ti_6_Al_4_V)	4420	0.56	7.2	5.8 × 10^5^

The equation for radiative flux given by Rosseland^68^ is applied in formula (5).

5 $${q}_{r}=-1.33\frac{{\sigma }^{*}}{{k}^{*}}{{\mathrm{\yen }}^{4}}_{\mathrm{y}},$$

where ${\sigma }^{*}$ signifies Stefan-Boltzmann constant and ${k}^{*}$ symbolizes the rate.

## The considered problem solve

Expressions (2)–(4) are BVP, as shown by likeness change, which converts the administered PDEs to ODEs. The formula's stream function is as follows:

6 $${\Lambda }_{1}={\psi }_{y}, {\Lambda }_{2}=-{\psi }_{x}.$$

The specified similarity quantities are

7 $$\lambda (x,y)=\sqrt{\frac{q}{{\nu }_{f}}}y, \psi (x,y)=\sqrt{{\nu }_{f}q}xf(\lambda ), \theta (\lambda )=(\mathrm{\yen }-{\mathrm{\yen }}_{\infty }){{(\mathrm{\yen }}_{w}-{\mathrm{\yen }}_{\infty })}^{-1}.$$

into Eqs. (2)–(4). We get

8 $$f^{\prime \prime \prime}+{R}_{1}{R}_{2}\left[ff^{\prime \prime}-{f}^{\prime 2}\right]-{P}_{b}f^{\prime}-{F}_{1}{F}_{2}{P}_{a}f^{\prime}=0,$$

9 $$\theta^{\prime \prime} \left(1+\frac{1}{{R}_{4}}{P}_{r}{N}_{\alpha }\right)+{P}_{r}\frac{{R}_{3}}{{R}_{4}}\left[f\theta^{\prime} -f^{\prime}\theta +\frac{{E}_{\alpha }}{{R}_{a}{R}_{c}}{f}^{\prime \prime 2}+\frac{{R}_{5}}{{R}_{3}}{P}_{a}{E}_{\alpha }{f}^{\prime 2}-{\Upsilon}_{\Lambda }\left({f}^{\prime 2}\theta -f^{\prime \prime} \theta -{f}^{2}{\theta }^{2}-ff^{\prime}\theta ^{\prime \prime} \right)\right]=0,$$

with

10 $$\left.\begin{array}{l}f(0)=S, f {^{\prime}}(0)=1+{\upchi }_{\Lambda }f{^{\prime}}{^{\prime}}(0), \theta {^{\prime}}(0)=-{B}_{\Lambda }(1-\theta (0))\\ f{^{\prime}}(\lambda )\to 0, \theta (\lambda )\to 0, as \lambda \to \infty \end{array}\right\}$$

Equation (2) is accurately confirmed. Previously, the sign ${^{\prime}}$ existed for demonstrating the derivatives regarding $\gamma$, see (Table 3).

**Table 3:** Description of the embedded constant parameters.

Symbols	Name	Formule	Default value
${\Upsilon}_{\Lambda }$	Relaxation time	${\Upsilon}_{\Lambda }=b\Upsilon$	0.1
${P}_{b}$	Porous media	${P}_{b}=\frac{{\nu }_{f}}{Lk}$	0.1
${P}_{r}$	Prandtl number	${P}_{r}$ =$\frac{{\nu }_{f}}{{\alpha }_{f}}$	6450
$\phi$	Volume fraction	–	0.18
${P}_{a}$	Magnetic parameter	${P}_{a}=\frac{{\sigma }_{f}{B}^{2}}{c{\rho }_{f}}$	0.1
$S$	Suction/injection parameter	$S=-{V}_{\Lambda }\sqrt{\frac{1}{{\nu }_{f} L}}$	0.4
${N}_{\alpha }$	Thermal radiation parameter	${N}_{\alpha }=5.33\frac{{\sigma }^{*}{\mathrm{\yen }}_{\infty }^{3}}{{\kappa }^{*}{\nu }_{f}(\rho {C}_{p}{)}_{f}}$	0.3
${B}_{\Lambda }$	Biot number	${B}_{\Lambda }=\frac{{h}_{\Lambda }}{{k}_{\Lambda }}\sqrt{\frac{{\nu }_{f}}{L}}$	0.2
${E}_{\alpha }$	Eckert number	${E}_{\alpha }=\frac{{U}_{w}^{2}}{({C}_{p}{)}_{f}({\mathrm{\yen }}_{w}-{\mathrm{\yen }}_{\infty })}$	0.2
${\chi }_{\Lambda }$	Velocity slip	${\chi }_{\Lambda }=\sqrt{\frac{L}{{\nu }_{f}}}{N}_{\Omega }$	0.3

Where ${R}_{1}{, R}_{2}, {R}_{3}$, ${R}_{4}$ and ${R}_{5}$ are given in Table 4

**Table 4:** Short expressions of embedded constant parameters.

Constants	Expression
${R}_{1}$	${R}_{1}=(1-{\phi }_{Co}{)}^{2.5}(1-{\phi }_{TA}{)}^{2.5}$
${R}_{2}$	${R}_{2}=(1-{\phi }_{TA})\left[(1-{\phi }_{Co})+{\phi }_{Co}\frac{{\rho }_{{p}_{1}}}{{\rho }_{f}}\right]+{\phi }_{TA}\frac{{\rho }_{{p}_{2}}}{{\rho }_{f}},$
${R}_{3}$	${R}_{3}=(1-{\phi }_{TA})\{(1-{\phi }_{Co})+{\phi }_{Co}\frac{(\rho {C}_{p}{)}_{{p}_{1}}}{(\rho {C}_{p}{)}_{f}}\}+{\phi }_{TA}\frac{(\rho {C}_{p}{)}_{{p}_{2}}}{(\rho {C}_{p}{)}_{f}},$
${R}_{4}$	${R}_{4}=\frac{{\kappa }_{hnf}}{{\kappa }_{f}}=\left[\frac{({\kappa }_{{p}_{2}}+2{\kappa }_{nf})-2{\phi }_{TA}({\kappa }_{nf}-{\kappa }_{{p}_{2}})}{({\kappa }_{{p}_{2}}+2{\kappa }_{nf})+{\phi }_{TA}({\kappa }_{nf}-{\kappa }_{{p}_{2}})}\right]\left[\frac{({\kappa }_{{p}_{1}}+2{\kappa }_{f})+{\phi }_{Co}({\kappa }_{f}-{\kappa }_{{p}_{1}})}{({\kappa }_{{p}_{1}}+2{\kappa }_{f})-2{\phi }_{Co}({\kappa }_{f}-{\kappa }_{{p}_{1}})}\right],$
${R}_{5}$	${R}_{5}=\frac{{\sigma }_{hnf}}{{\sigma }_{f}}=\left[1+\frac{3\left(\frac{{\phi }_{Co}{\sigma }_{{p}_{1}}+{\phi }_{2}{\sigma }_{{p}_{2}}}{{\sigma }_{f}}-({\phi }_{Co}+{\phi }_{TA})\right)}{(\frac{{\phi }_{Co}{\sigma }_{{p}_{1}+{\phi }_{TA}{\sigma }_{{p}_{2}}}}{({\phi }_{Co}+{\phi }_{TA}){\sigma }_{f}}+2)-(\frac{{\phi }_{Co}{\sigma }_{{p}_{1}}+{\phi }_{TA}{\sigma }_{{p}_{2}}}{{\sigma }_{f}}-({\phi }_{Co}+{\phi }_{TA}))}\right].$

The non-dimensional skin friction $({C}_{f})$, Nusselt number $(N{u}_{x})$ and Entropy generation $\left({N}_{g}\right)$ expressions are postulated as

11 $${C}_{f}R{e}_{x}^\frac{1}{2}=\frac{f{^{\prime}}{^{\prime}}(0)}{{R}_{1}}, N{u}_{x}R{e}_{x}^{-\frac{1}{2}}=-\frac{{k}_{hnf}}{{k}_{f}}\left(1+{N}_{\alpha }\right)\theta {^{\prime}}(0),$$

$${N}_{G}={R}_{\Gamma }\left[{R}_{4}(1+{N}_{\alpha }){\theta {^{\prime}}}^{2}+\frac{1}{{R}_{1}}\frac{{B}_{\Gamma }}{\Gamma }\left({f{^{\prime}}{^{\prime}}}^{2}+({P}_{b}+{R}_{1}{R}_{5}{P}_{a}){f{^{\prime}}}^{2}\right)\right].$$

where ${C}_{f}$ represents the coefficient of drag force. $R{e}_{x}=\frac{{u}_{w}x}{{\nu }_{f}}$ is local $Re$ according to the elongated velocity ${u}_{w}(x)$. Additionally, R_Γ denotes the Reynolds value, B_Γ the Brinkman value, and the non-dimensional temperature differential.

## Galerkin finite element technique

**Figure 2:**
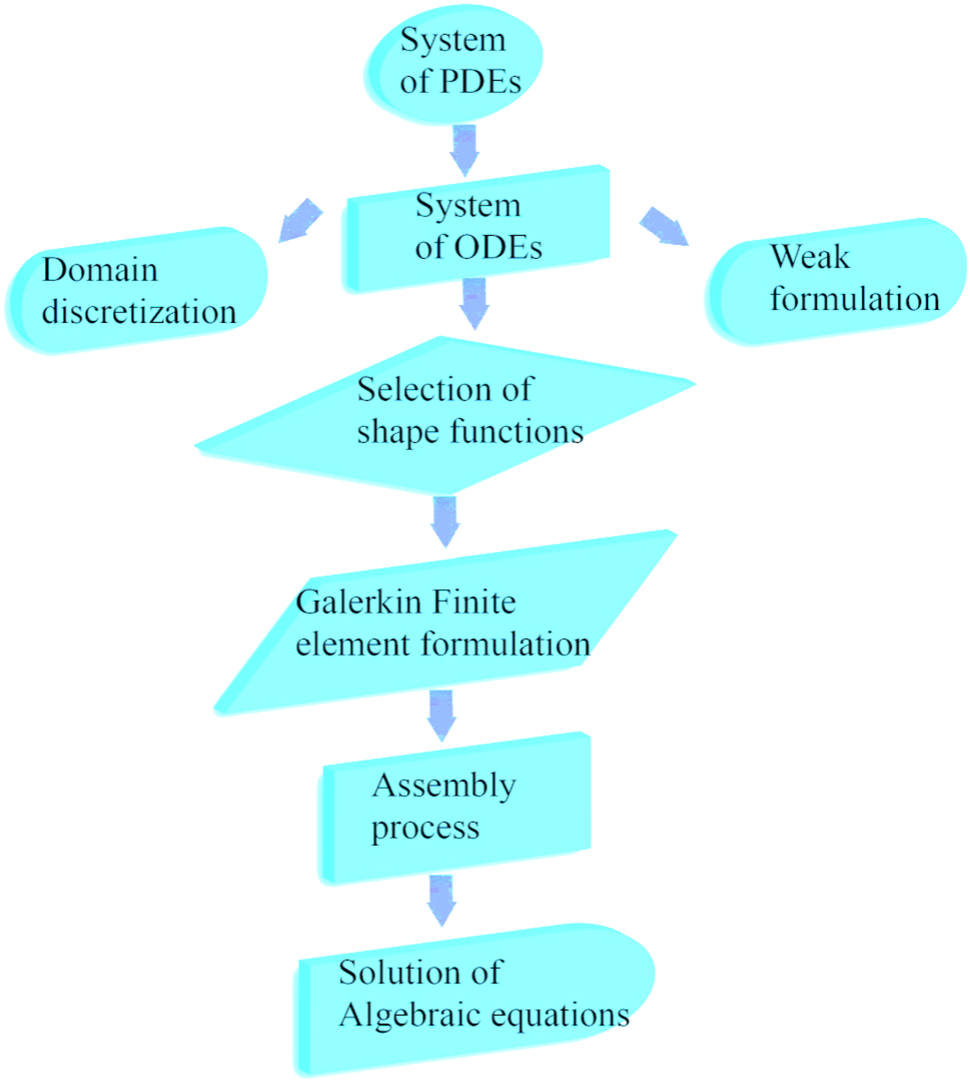
Flow chart of G-FEM.

The corresponding boundary constraints of the present system were computationally simulated using FEM. FEM is based on the partitioning of the desired region into components (finite). FEM^69^ is covered in this section. The finite element method's flowchart is shown in Fig. 2. Numerous computational fluid dynamics (CFD) problems have been addressed using this technique; the advantages of doing so are covered in more detail below.

Stage I: Weak form is derived from strong form (stated ODEs), and residuals are computed.
Stage II: To achieve a weak form, shape functions are taken linearly, and FEM is used.
Stage III: The assembly method is used to build stiffness components, and a global stiffness matrix is created.
Stage IV: Using the Picard linearizing technique, an algebraic framework (nonlinear equations) is produced.
Stage V: Algebraic equations are simulated utilizing appropriate halting criterion through 10(-5) (supercomputing tolerances).

$$\left|\frac{{\lambda }_{i+1}-{\lambda }_{i}}{{\lambda }^{i}}\right|<{10}^{-5}.$$

Further, The Galerkin finite element technique's flow chart is depicted in Fig. 2.

## Verification of code

Heat transfer coefficients from existing methods were compared to findings that had been supported by earlier research to assess the validity of the computational method^70^. Table 5 displays a comparison of the outcomes of the current study with those of earlier investigations. The outcomes of recent investigations are comparable and remarkably accurate.

**Table 5:** Evaluation regarding the values of $-{\theta }{{^{\prime}}}(0)$ with ${P}_{r}$, with fixed $\phi =0$, ${\phi }_{hnf}=0$, ${E}_{\alpha }=0$, ${P}_{b}=0$, ${\upchi }_{\Lambda }=0$, ${N}_{\alpha }=0$, $S=0$ and ${B}_{\Lambda }\to 0$.

${P}_{r}$	Abu-Hamdeh et al.^70^	This study
0.72	0.8087618	0.8087225
1.0	1.0000000	1.0000000
3.0	1.9235742	1.92341053
7.0	3.0731465	3.0731389
10	3.7205542	3.7205678

## Main findings and their descriptions

This section delves into the influence of a few key physical parameters, such as the velocity slip parameter $({\chi }_{\Lambda })$, thermal radiation parameter $\left({N}_{\alpha }\right)$, Biot number $\left( {B}_{\Lambda }\right)$, volume fraction parameter $\left(\phi , {\phi }_{hnf}\right),$ porous media parameter $\left({P}_{b}\right),$ Eckert number $\left({E}_{\alpha }\right)$, and Brinkmann's number $\left({B}_{\Gamma }\right)$ upon temperature $\left(\theta \left(\lambda \right)\right),$ velocity (*f*′(*λ*)) and entropy generation $\left({N}_{G}\left(\lambda \right)\right)$ fields. The nanofluid particles Cu and Ti_6_Al_4_V are composed of water. The solid and dashed lines are respectively plotted for Co-H_2_O and Ti_6_Al_4_V-Co/ H_2_O.

**Figure 3:**
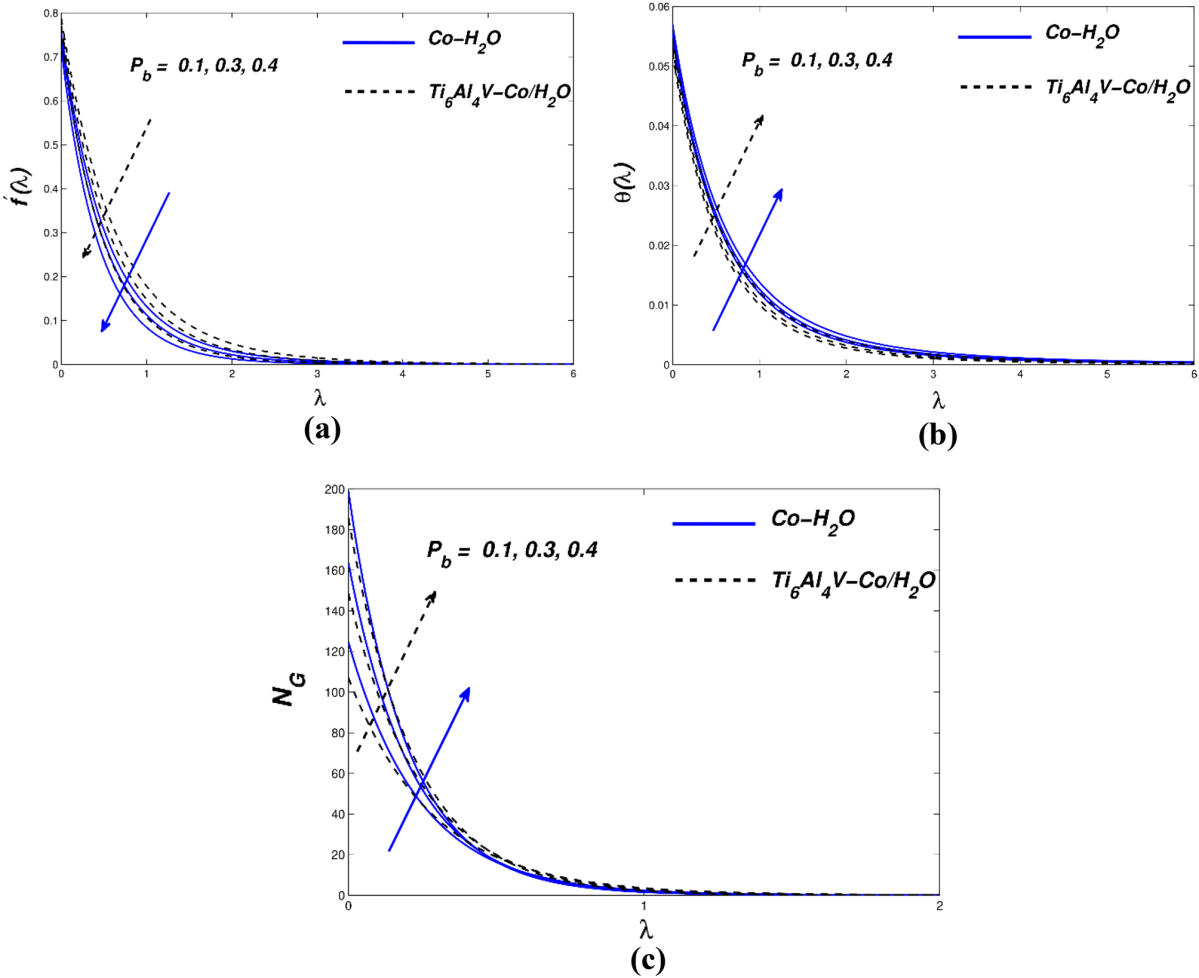
(**a**) ${f}{^{\prime}}(\lambda )$ (**b**) $\theta (\lambda )$ and (**c**) ${N}_{G}$ with diverse ${P}_{b}$ values.

**Figure 4:**
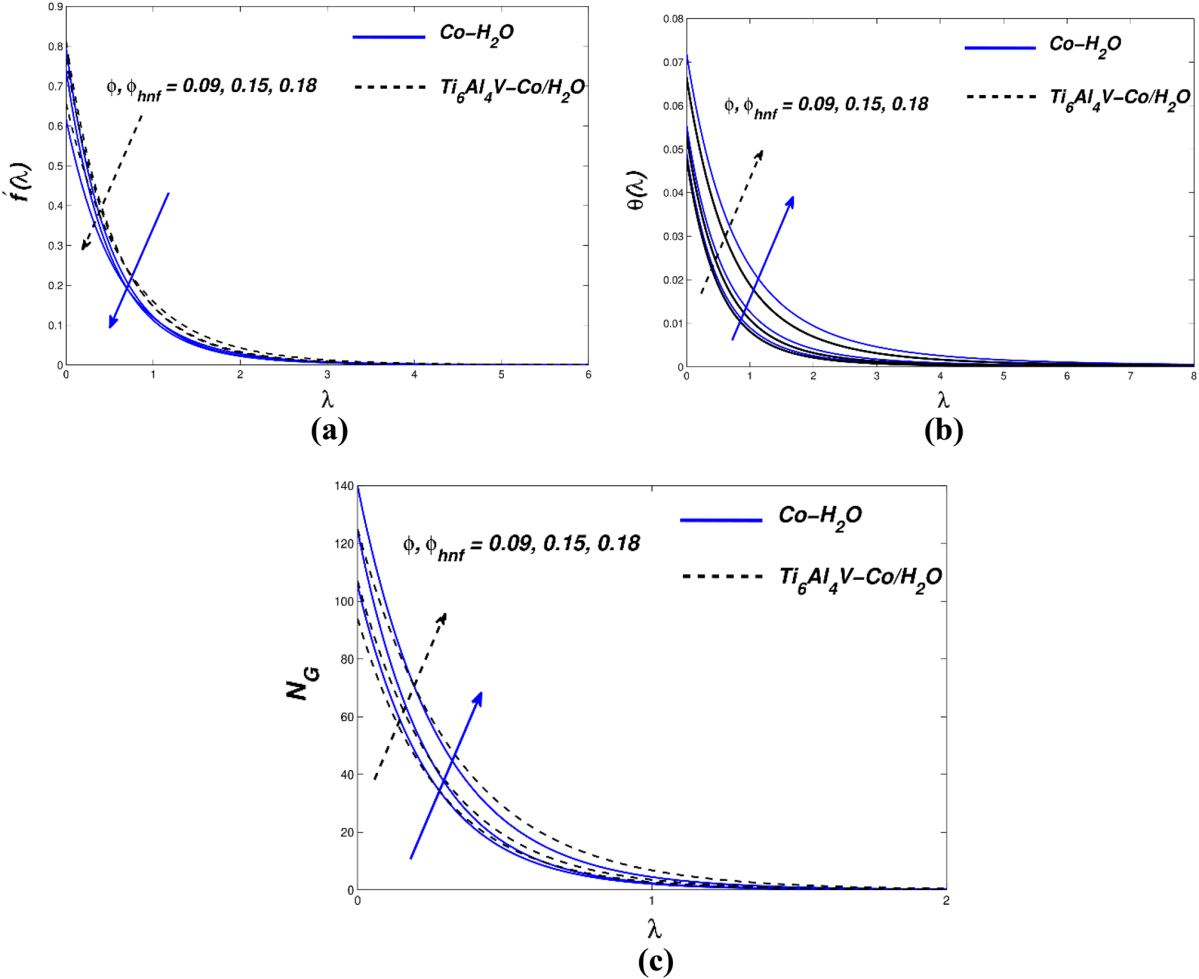
(**a**) ${f}^{{\prime}}(\lambda )$ (**b**) $\theta (\lambda )$ and (**c**) ${N}_{G}$ with diverse $\phi$ as well as ${\phi }_{hnf}$ values.

**Figure 5:**
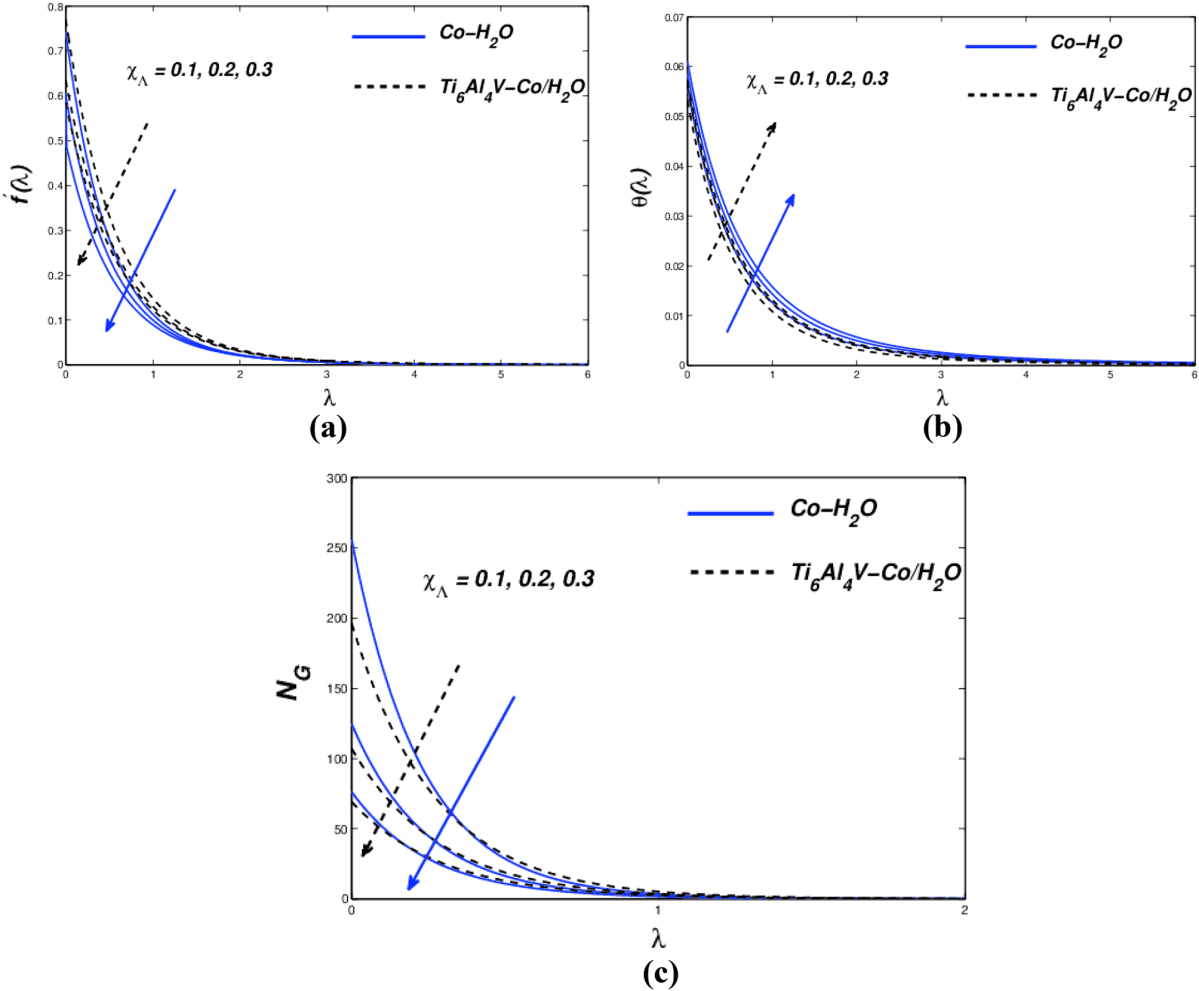
(**a**) ${f}^{{\prime}}(\lambda )$ (**b**) $\theta (\lambda )$ and (**c**) ${N}_{G}$ with diverse ${\chi }_{\Lambda }$ values.

Figure 3a–c illustrate how the permeability parameter (${P}_{b}$) affects the flow, temperature, and entropy distribution of hybrid nanofluids. As seen in Fig. 3a, the permeability parameter (${P}_{b}$) affects the flow distribution. A plate-like surface is created when a hybrid non-liquid flow channel enters a porous material and draws velocity along it. As the porosity grows sufficiently, there are extremely few nanoparticle collisions and less heat output. Viscosity reduces the flow rate by modulating buoyancy. The inverse response is shown in the graphic. As shown in Fig. 3b, increasing density causes the flow temperature to rise. Figure 3c displays *N*_*G*_ vs. (${P}_{b}$) entropy generation. In this case, the surface value of (*N*_*G*_) grows but the value of (P b) declines as the distance from the surface increases. A major temperature differential at the surface causes entropy to increase. Consequently, a high value of the permeability of the porous medium may present a technique for modifying the spin coating flow parameters in industrial applications. It is also believed that improved permeability and larger pore spaces promote better nanoparticle precipitation, which reduces friction at the sheet surface. Figure 4a–c show how flow velocity, temperature field, and entropy affect the sensitivity of nanomolecular size. The five nanofluid coefficients that have an impact on volume fraction are given in Table 4 as a result of the creation of the Tiwari-Das model. The fluid's velocity drops as the volume ratio of nanoparticles rise (Fig. 4a). These flows are impeded by the increase in magnetic viscosity that occurs with a velocity decrease. The bigger the volume fraction of nanoparticles, the faster the temperature rises. Due to improved heat transmission between hybrid and conventional fluid–solid suspension nanoparticles, the fluid binding force inside the fluid–solid suspension system is reduced. According to the flow distribution, the nanofluid material has a high conductivity coefficient and convectional heat transfer. Therefore, nanofluid heat transfer is the driving force behind the most significant industrial and technological advancements of our time. Therefore, Fig. 4b thermal improvement is supported. Nanoparticles are added to boost thermal boundary expansion through ballistic impacts, which improves heat conduction and liquid viscosity. When compared to Ti_6_Al_4_V-Co/H_2_O nanoparticles, Co-H_2_O nanoparticles control heat transport in the examined base fluid. Figure 4c shows the consequence of varying the nanomaterial term on entropy generation. There was a clear difference in the behavior of the curves when increasing the volume percentage and approaching the strain wall. The total volume fraction parameter increased due to faster heat transfer and enhanced entropy generation in the hybrid nanofluidic zone. The velocity-slip parameter (${\chi }_{\Lambda }$) affects the velocity, temperature, and entropy formation of the Fig. 5a–c. We examine and assess the sensitivity of strain parameters deriving from boundary conditions using typical hybrid momentum distributions in nanofluids. The liquid slows down because its viscosity rises quadratically with the velocity divergence (Fig. 5a). As a result, conventional and hybrid nanofluids have lower profiles in the thermal boundary layer (Fig. 5b).

**Figure 6:**
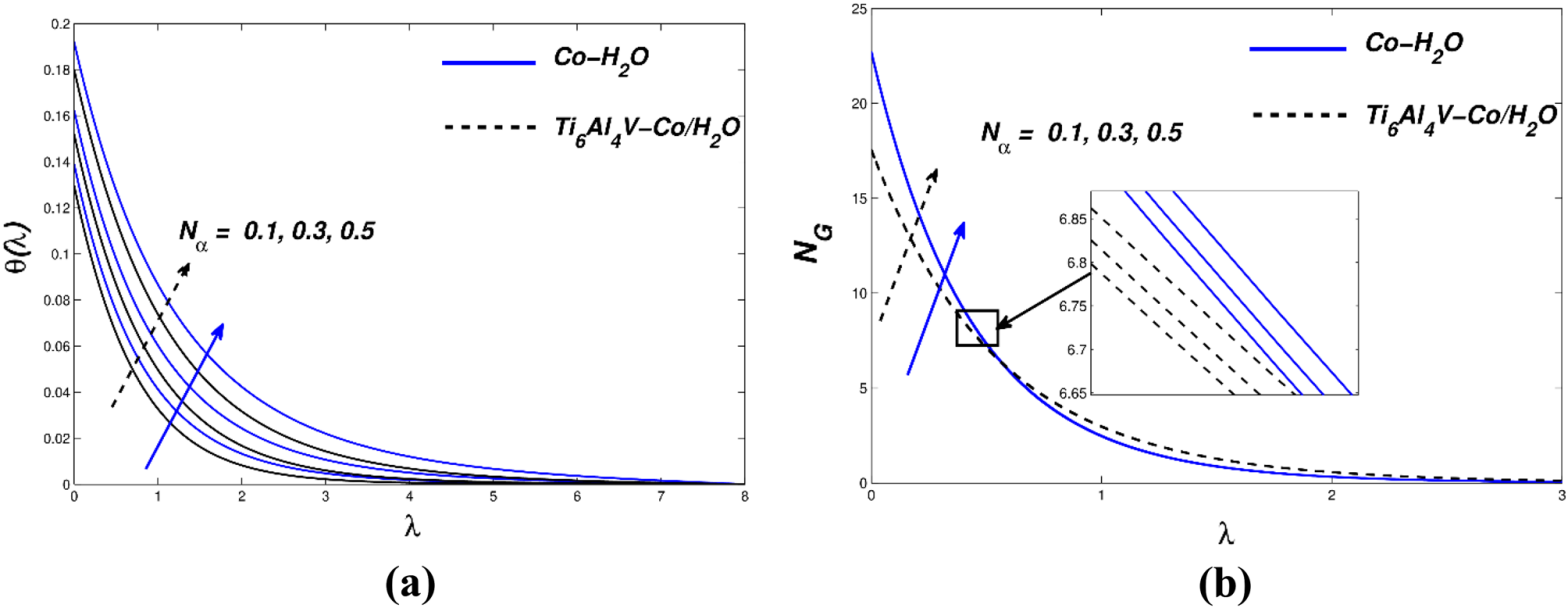
(**a**) $\theta (\lambda )$ and (**b**) ${N}_{G}$ with diverse ${N}_{\alpha }$ values.

**Figure 7:**
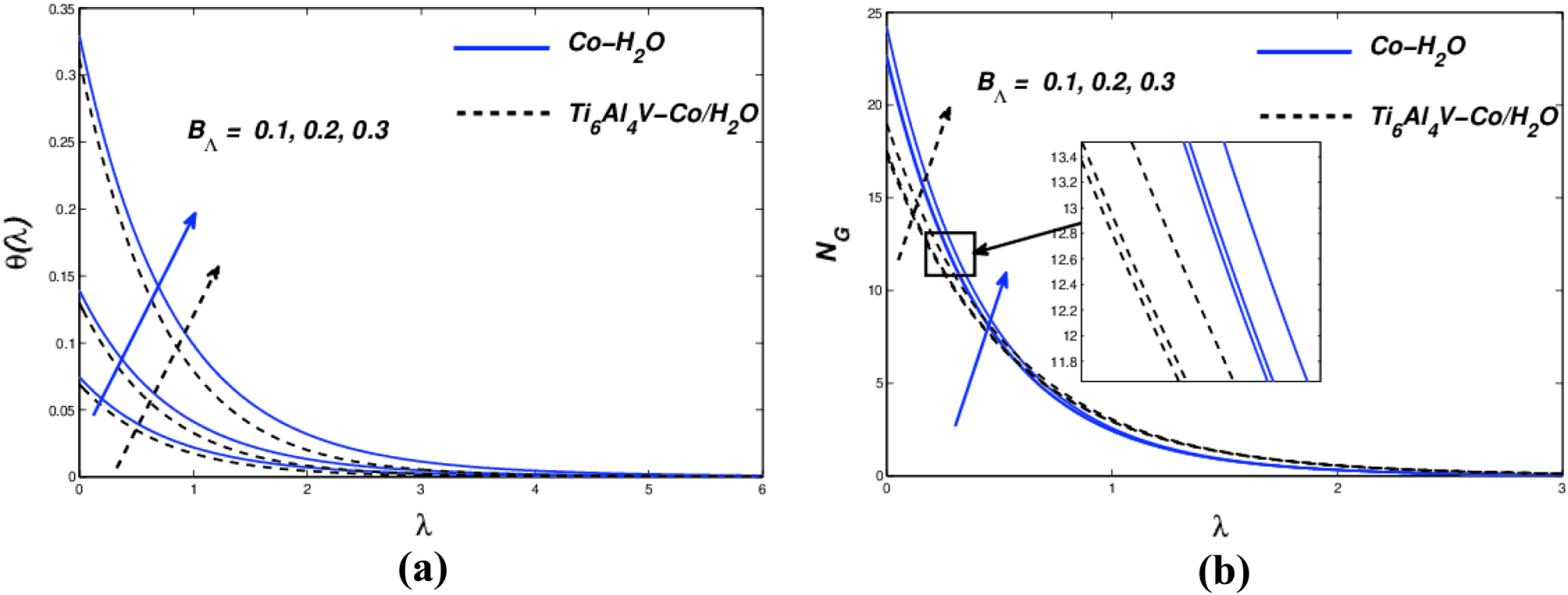
(**a**) $\theta (\lambda )$ and **(b)** ${N}_{G}$ with diverse ${B}_{\Lambda }$ values.

**Figure 8:**
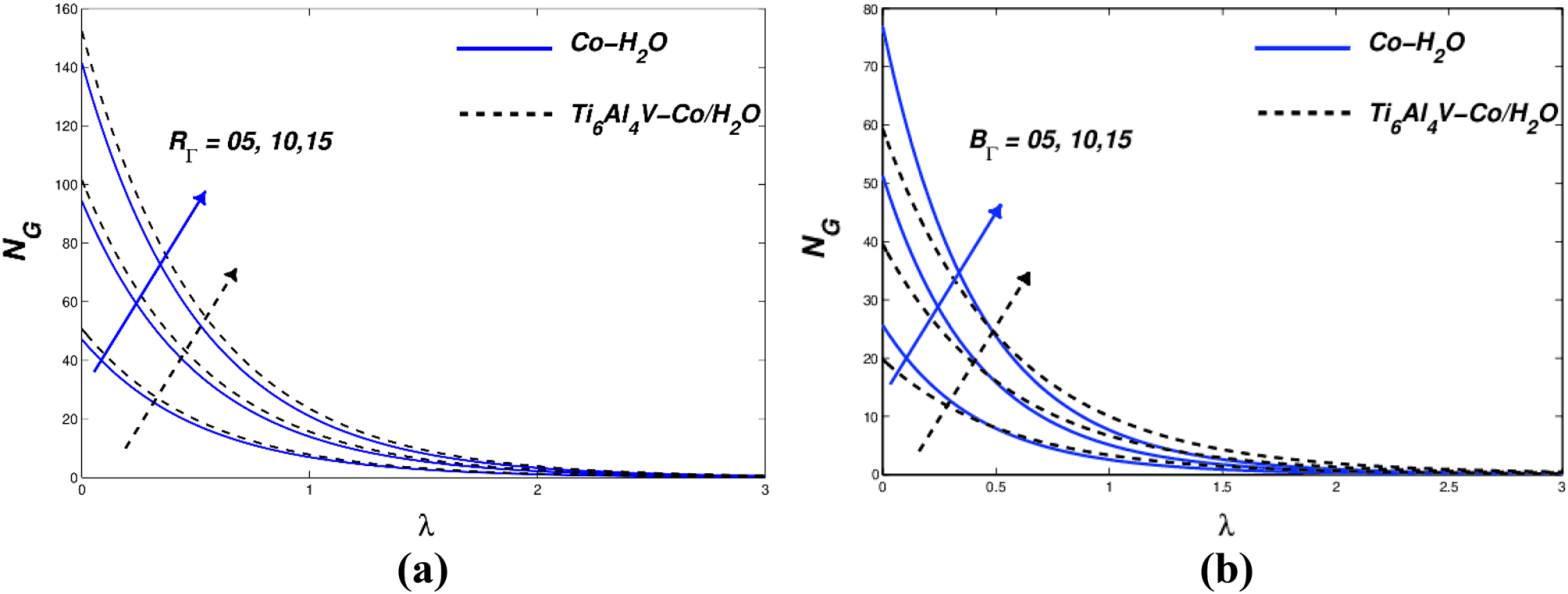
(**a**) Variations in entropy concerning ${R}_{\Gamma }$ **(b)** Variations in entropy concerning ${B}_{\Gamma }$.

Even if the wall velocity parameter has significant slip velocity values, it restricts collisions with molecular diffusion. When more nanoparticles are added to various mediums, the simultaneous effects of thermal convection, diffusion, and kinematic viscosity are involved. In Fig. 5c, *N*_*G*_ is shown as a deviation from the variety of entropy produced. (${\chi }_{\Lambda }$) modifications throughout the plate are impervious (powerful diminution) due to the fact they're away from the plate, consistent with the innovative amount graph of *N*_*G*_ and (${\chi }_{\Lambda }$). Due to the slip condition inside the velocity implementation, entropy suggests an inventive reduction in entropy creation. The dimensionally inactive radiative parameter (${N}_{\alpha }$) is shown in a variety of values in the temperature arc of Fig. 6a. To increase the temperature profile of the flow, the radiation parameter (${N}_{\alpha }$) must be intensified. The temperature of the nanofluid rises as (${N}_{\alpha }$) rises. Although the thermal radiation parameter is more important, radiant flux still provides thermal energy to the process. The boundary layer is maintained by this temperature. The Ti_6_Al_4_V-Co/ H_2_O hybrid nanofluid and Co-H_2_O nanofluid are shown in Fig. 6b together with the influence of entropy generation. Figure 6b also depicts the fluid dynamics of the radiation parameter (${N}_{\alpha }$) for both nanofluids. As can be seen, differing valences of the radiation parameters (${N}_{\alpha }$) drive entropy production. Therefore, the radiation parameter greatly influences the entropy distribution of stretched porous devices. Figure 7a displays the thermal behaviour for various Biot numbers (${B}_{\Lambda }$). The linear response for Co-H2O and Ti_6_Al_4_V-Co/ H_2_O nanoparticles is predicted to increase (${B}_{\Lambda }$). In the thermal thin state, which denotes that the body temperature is typically uniform, a low Biot number (${B}_{\Lambda }$) is significant (on the nanopolymer surface). Higher (${B}_{\Lambda }$) values denote dense thermal patches with irregular temperature domains. Figure 7a depicts how *N*_*G*_ behaves when the Biot number (${B}_{\Lambda }$) value rises. A steady increase in surface variance is less sensitive than a gradual drop away from the surface, as seen in Fig. 7b. H. A small but noticeable escalation along the wall of the stretch area. (${B}_{\Lambda }$) Evolution further away from the plate causes a decrease in entropy generation. From the graph, we can see that *N*_*G*_ is very sensitive to surface and small changes. For both kinds of nanofluids, entropy generation profiles as a function of the Reynolds number (${R}_{\Gamma }$) are displayed in Fig. 8a. It has been found that better (${R}_{\Gamma }$) has an impact on entropy. When the frictional effect is reversed, raising (${R}_{\Gamma }$), the entropy sketch is more pertinent. The difference between the *N*_*G*_ and ${B}_{\Gamma }$ values in Fig. 8b demonstrate that entropy production rises as the Brinkmann number (${B}_{\Gamma }$) rises. The Brinkmann number (${B}_{\Gamma }$) was created to research the negative impacts of liquids as a result. Friction is the primary contributor to the creation of entropy, according to the Brinkmann number (${B}_{\Gamma }$). This result shows that the Reynolds number and Brinkmann number of Ti_6_Al_4_V-Co/H_2_O nanoparticles are substantially higher than those of Co-H_2_O nanoparticles.

Table 6 is planned to display the control of various sundry dimensionless factors appear during numerical recreation of the problematic.

**Table 6:** Values of ${C}_{f}R{e}_{x}^{1/2}$ and ${Nu}_{x}R{e}_{x}^{-1/2}$ for ${P}_{r}=$ 6.2.

${P}_{a}$	${P}_{b}$	$\phi$	${\phi }_{TA}$	${\chi }_{\Lambda }$	${E}_{\alpha }$	${N}_{\alpha }$	${B}_{\Lambda }$	${C}_{f}R{e}_{x}^\frac{1}{2}$ Co-H_2_O	${C}_{f}R{e}_{x}^\frac{1}{2}$ Ti_6_Al_4_V-Co/H_2_O	${N}_{u}R{e}_{x}^{\frac{-1}{2}}$ Co-H_2_O	${N}_{u}R{e}_{x}^{\frac{-1}{2}}$ Ti_6_Al_4_V-Co/H_2_O
0.1	0.1	0.18	0.09	0.3	0.2	0.3	0.2	2.3523	3.4241	1.2610	2.2736
0.6								2.3736	3.4666	1.2361	2.3564
1.6								2.4052	3.5068	1.2024	2.3137
	0.1							2.3523	3.4241	1.2610	2.2736
	0.3							2.3811	3.4323	1.2941	2.3067
	0.4							2.4125	3.4636	1.3164	2.3445
		0.09						2.2947	–	1.2289	–
		0.15						2.3125	–	1.2461	–
		0.18						2.3523	–	1.2610	–
			0.0					–	2.2947	–	1.2289
			0.06					–	3.4056	–	2.2927
			0.09					–	3.4241	–	2.2736
				0.1				2.4135	3.4823	1.2035	2.2063
				0.2				2.3823	3.4566	1.2369	2.2350
				0.3				2.3523	3.4241	1.2610	2.2736
					0.1			2.3412	3.6130	1.2323	2.2563
					0.2			2.3523	3.4241	1.2610	2.2736
					0.3			2.3523	3.4241	1.2895	2.2884
						0.1		2.3523	3.4241	1.2023	2.2331
						0.3		2.3523	3.4241	1.2610	2.2736
						0.5		2.3523	3.4241	1.2968	2.3061
							0.1	2.3523	3.4241	1.2426	2.2358
							0.2	2.3523	3.4241	1.2610	2.2736
							0.3	2.3523	3.4241	1.3471	2.2948

## Concluding remarks

Entropy creation, irreversibility propagation, fluid flow, and heat transfer in an electrically conducting Newtonian hybrid nanofluid across a stretching sheet exposed to slip and convective boundary conditions have all been quantitatively described in the current research. The solid volume fraction has been explored using a modified version of Tiwari and Das's nanofluid model of the Co-H_2_O and Ti_6_Al_4_V-Co/H_2_O nanoparticles. Graphic analysis and extensive discussion of the physical behavior of the non-dimensional boundary layer distributes show how the unique factors affect them. Thus, from the present analysis, the under-listed concluding remarks are obtained:

- Along the far stream, the velocity field is reduced for the upsurging porosity $({P}_{b})$, volume fraction $(\phi , {\phi }_{hnf}),$ and velocity slip $({\chi }_{\Lambda })$.
- The temperature distribution is affected by most of the physical quantities, which denotes that nanofluids have a high heat exchange rate. This property helps control the temperature during spin coating processes.
- The entropy profile against the porosity term $({P}_{b})$, volume fraction $(\phi , {\phi }_{hnf})$ and radiation parameter $({N}_{\alpha })$, Biot number $({B}_{\Lambda })$ explore dual behavior.
- Remarkable change in frictional force factor for Co-H_2_O nanofluid and Ti_6_Al_4_V-Co/H_2_O hybrid nanofluids can be seen, compared to the Nusselt number coefficient for the porosity and volume fraction.

The FEM could be applied to a variety of physical and technical challenges in the future^71–76^.

## Data Availability

All data generated or analysed during this study are included in this published article.
